# Diverse alveolate infections of tadpoles, a new threat to frogs?

**DOI:** 10.1371/journal.ppat.1008107

**Published:** 2020-02-13

**Authors:** Aurelie Chambouvet, Vanessa Smilansky, Miloslav Jirků, Marcos Isidoro-Ayza, Sarah Itoïz, Evelyne Derelle, Adam Monier, David J. Gower, Mark Wilkinson, Michael J. Yabsley, Julius Lukeš, Thomas A. Richards

**Affiliations:** 1 CNRS, Univ Brest, IRD, Ifremer, LEMAR, Plouzané, France; 2 Biosciences, Living Systems Institute, University of Exeter, Exeter, United Kingdom; 3 Institute of Parasitology, Biology Centre, Czech Academy of Sciences, České Budějovice (Budweis), Czech Republic; 4 Department of Pediatrics, School of Medicine and Public Health, University of Wisconsin, Madison, Wisconsin, United States of America; 5 Department of Life Sciences, Natural History Museum, London, United Kingdom; 6 Warnell School of Forestry and Natural Resources and the Southeastern Cooperative Wildlife Disease Study, Department of Population Health, College of Veterinary Medicine, University of Georgia, Athens, Georgia, United States of America; 7 Faculty of Sciences, University of South Bohemia, České Budějovice (Budweis), Czech Republic; 8 Department of Zoology, University of Oxford, Oxford, United Kingdom; Children's Hospital of Philadelphia, UNITED STATES

## Frogs are in decline

Amphibians are one of the most threatened major groups of animals, with decline in amphibian populations often cited as support for the claim that we are witnessing a mass extinction event [1]. The following causes of amphibian decline have been suggested: 1) invasive species causing ecosystem change, 2) overexploitation of natural environments, 3) changes in land use, 4) global environmental change, such as global warming, 5) increased use of pesticides and other polluting chemicals, and 6) the emergence and/or spread of infectious diseases [1–3]. We need to consider all of these factors if we are to understand amphibian decline and plan conservation strategies accordingly.

**Fig 1:**
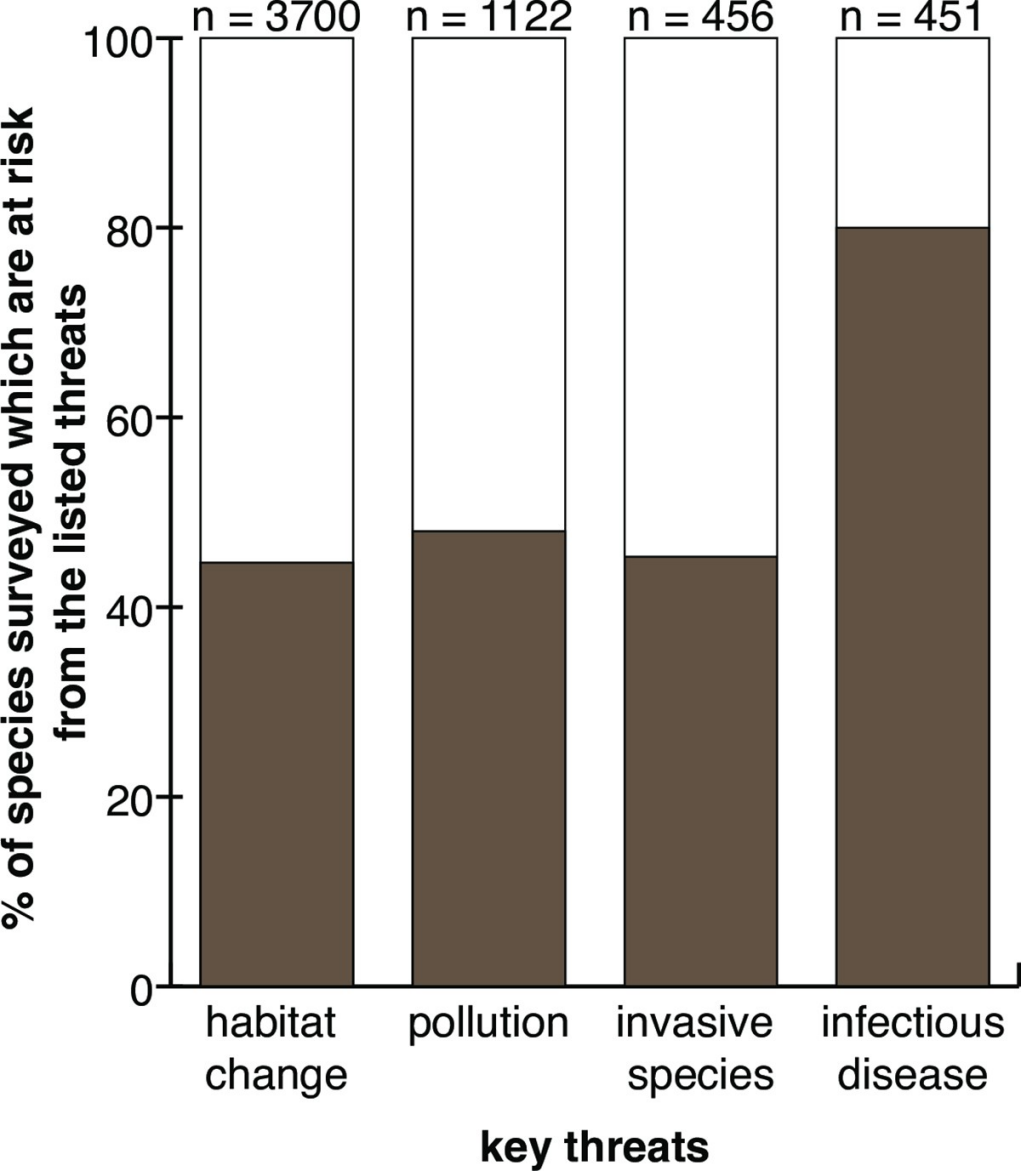
Graph illustrating key threats to amphibians. *Adapted from Chanson and colleagues (2008)* [2].

**Fig 2:**
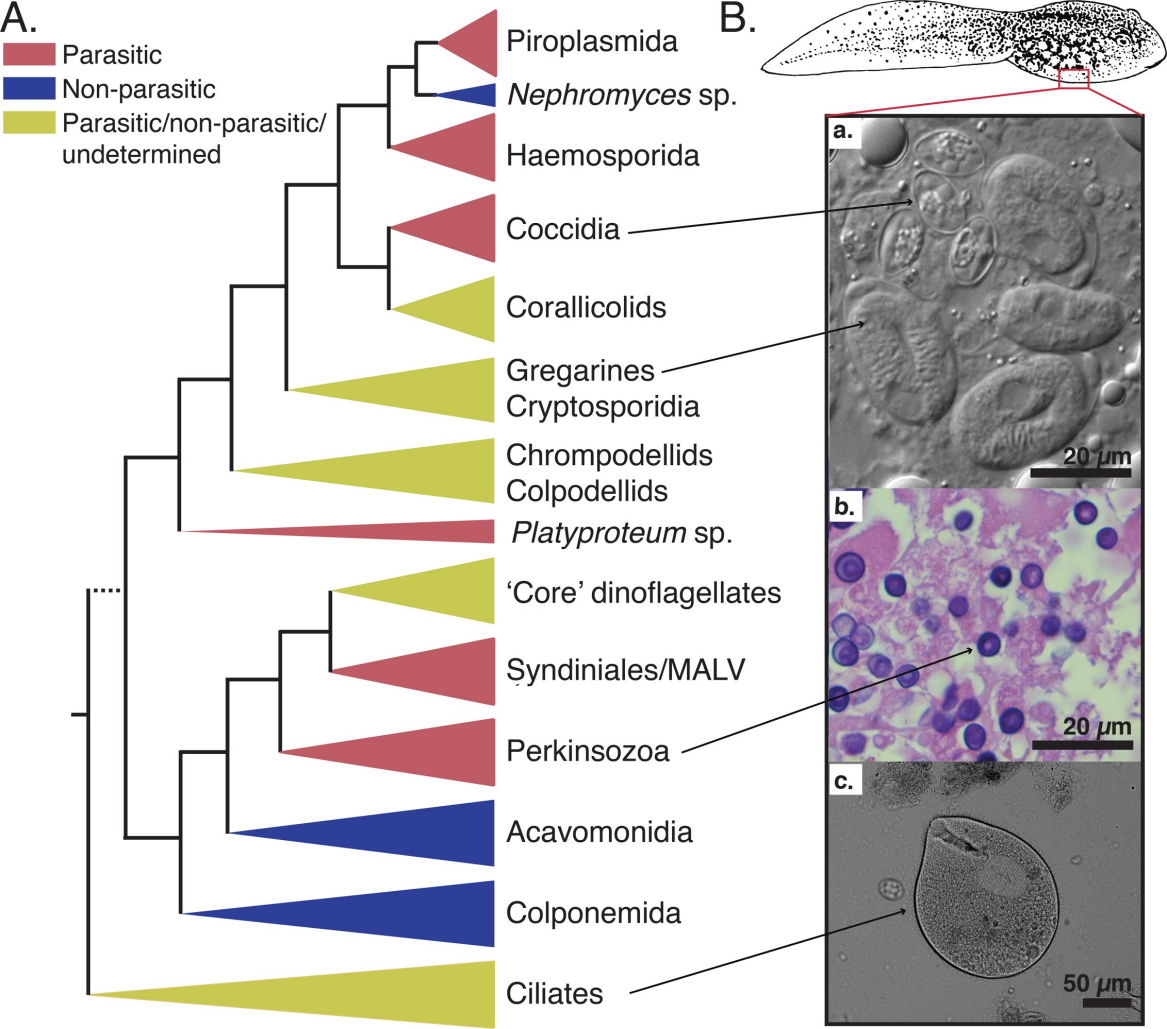
Schematic tree of the Alveolata superphylum illustrated with some examples of tadpole infectious agents. A. Schematic diagram of the relationships among the three main lineages of the Alveolata superphylum based on rDNA phylogeny (not to scale), with parasitic and nonparasitic lineages indicated. Dotted line for the basal branch is hypothetical. Adapted from Mickhailov and colleagues (2014) [15]. B. Micrographs of tadpole liver and intestine samples infected by protists belonging to the Alveolata superphylum. a. Light microscopy of macrophages containing several oocysts of both *Nematopsis temporariae* (Gregarines) and *Goussia noelleri* (Coccidia) from tadpole liver samples of *Rana dalmatina*, fresh mounts, NIC [6] b. Histological section of infected liver tissue samples from a River frog (*Rana heckscheri)* tadpole mass mortality event in southwestern Georgia (USA) in 2006, stained with hematoxylin–eosin (Yabsley, unpublished). c. Light microscopy of putatively commensal ciliate *Balantidium* sp. from tadpole intestine samples of *Bombina bombina*, fresh mounts, NIC (Jirků, unpublished). *The tadpole drawing is a free public domain vector cliparts (available on* *www*.*clker*.*com**)*. NIC, Nomarski interference contrast.

Importantly, infectious-disease–associated decline is cited as a major factor affecting amphibian species categorized as threatened by the International Union for Conservation of Nature (IUCN) Red List (Fig 1). This may be because these species have been studied closely—so disease threats are identified and tracked—or it could be because disease is indeed a key threat for many amphibian groups in decline. However, infectious diseases are difficult to study in amphibians, because the underlying causes of susceptibility to infection are often difficult to pinpoint, the identities of infectious agents or the nature of virulence is unclear, and adequate sampling of populations and the associated disease biogeography is challenging. Recent work has consistently demonstrated that a wide range of protists of the superphylum Alveolata infects the tissues of larval amphibians [4–6]. The alveolates include a diversity of forms (Fig 2A)—for example, Apicomplexa, chrompodellids, Perkinsozoa, dinoflagellates, and Ciliophora (i.e., ciliates). In some cases, a link with disease has been identified, although formal confirmation equivalent to fulfillment of Koch’s postulates [7] is lacking. Here, we discuss the diversity and nature of these infectious agents and outline future research questions.

## What do we know about emerging diseases in amphibian populations?

Emerging infectious diseases (EIDs) are defined as newly identified diseases in previously uninfected populations or infectious diseases demonstrating a rapid increase in incidence, virulence, or geographical range [1–3]. Over the last 10 years, EIDs have increasingly been identified as an important cause of amphibian population declines with two groups of parasites identified as major threats: chytrid fungi (*Batrachochytrium dendrobatidis* [*Bd*] and *B*. *salamandrivorans* [*Bsal*]) and viruses of the genus *Ranavirus* [2,3,8]. *Bd* and *Bsal* infect amphibian skin. In immunologically naïve amphibians, the infection develops into a clinical disease (chytridiomycosis) with typical symptoms including hyperkeratosis, epidermal hyperplasia, and ulcers. This disease leads to altered host osmoregulation, causing cardiac arrest. Chytridiomycosis has been diagnosed in a wide range of amphibians (>500 species), including members of all three extant amphibian orders, Anura, Caudata (salamanders and newts), and Gymnophiona (caecilians) [3,9,10]. Although both *Bd* and *Bsal* likely originated in Asia [11], it has been hypothesized that their recent spread has been facilitated by humans [3,11]. Today, *Bd* occurs nearly worldwide, with mass mortality events identified in Australia, Europe, and Central and North America [3].

The ranaviruses are members of the Iridoviridae family of double-stranded DNA viruses. These viruses have been detected with a near global distribution, and associated mass mortality events have been reported from the Americas, Europe, and Asia [8]. While chytridiomycosis and Ranavirus infections have been extensively documented for adult amphibians, understanding of these and other diseases in the larval phase of an amphibian life cycle (i.e., tadpoles) is limited because dead or diseased tadpoles are often not collected for postmortem assessment. Other infections of amphibians include, for example, Myxozoan Cnidarian parasites, Microsporidian protists, and necrotizing hepatitis virus [12]. Although such groups also deserve further study, they are not the focus of this article.

## Are larval amphibians—Tadpoles—Unique hosts for alternative and cryptic infections?

Yes, larval amphibians have a distinct and reduced immune function compared to adults [13] and often live in different environments, thus they can be subject to distinct infectious disease ecologies. Although tadpoles, froglets, and adults are immuno-competent, tadpoles have the weakest adaptive immunity. This is evident by having fewer antibody classes, reduced B and T lymphocyte function, inconsistent displays of major histocompatibility complex class I protein, and a limited switch from Immunoglobulin M (IgM) to Immunoglobulin Y (IgY) [13]. Tadpoles therefore rely on an innate immune system of phagocytic macrophage cells that provide rapid and nonspecific protection against microbial infections, and it can therefore be postulated that they are more susceptible to parasitic infections than adults [13].

Only a few studies have documented the difference in susceptibility between different life stages of biphasic anurans (frogs with a lifecycle composed of two life cycle stages). *Bd* infects the keratinized mouthparts of the tadpoles, but chytridiomycosis symptoms do not manifest until after metamorphosis [14]. In contrast, ranaviruses have been found to infect and cause mortalities in all life cycle stages of the amphibians studied [8].

## What alveolate protists infect amphibian larvae?

The alveolate protists include a huge diversity of microbial forms and functional types such as phototrophs, bacterial grazers, and intracellular parasites (Fig 2A). These include diverse parasites of vertebrates and invertebrates and a range of parasites that infect marine and freshwater microbial eukaryotes [15]. A growing body of data has also shown that three phylogenetically distinct groups of alveolates infect internal organs of tadpoles: perkinsozoans, gregarines, and Coccidia. These parasites preferentially colonize liver tissues, forming intracellular infections of erythrocytes and macrophages, implying an infection that is detrimental to core physiology and/or immune function. However, this may—in-part—be an artifact of sampling, because different tadpole tissue types have yet to be sampled thoroughly. In all three alveolate lineages that infect tadpole livers, the life cycle of these infectious agents is not known and Koch’s postulates remain untested, so it is unclear if these infections represent disease-causing associations or if tadpoles represent an intermediate or definitive host of these parasites. In all three lineages, the tadpole-infecting alveolates are phylogenetically closely related to known parasites. In addition to these alveolates, which are known to branch with parasites, there are numerous ubiquitous, putatively commensal protists that inhabit the lumina of gastro-intestinal tracts of tadpoles—including, for example, extra- or epi-cellular alveolates such as: *Balantidium*, *Nyctotherus*, and *Trichodina* ciliates (Fig 2B) and *Opalina* stramenopiles.

## Do any of these alveolates cause severe disease?

In 2007, Davis and colleagues reported a large-scale mortality event in a population of Southern leopard frog tadpoles (*Lithobates sphenocephalus*) in a pond in Northeast Georgia, United States of America (USA) [4]. Infected tadpoles demonstrated lethargic swimming with recurrent abnormalities, including abdominal distension, subcutaneous oedema, cutaneous erythema and petechia, or patchy pale discoloration of the skin [4,16]. High densities of an initially unknown protist within the liver tissues (Fig 2B) were observed. Analysis of partial 18S rRNA gene sequences indicates that these protists lie within in a clade with members of the phylum Perkinsozoa (also known as perkinsids or Perkinsea) [17]. Perkinsozoa were traditionally thought of as a marine group that infects molluscs or dinoflagellate microalgae [17]. Indeed, marine members of this group have been classified as “emerging disease parasites” and the World Organisation for Animal Health has included the bivalve parasites *Perkinsus marinus* and *P*. *olseni* in the list of notifiable diseases (http://www.oie.int/en/animal-health-in-the-world/oie-listed-diseases-2019/). Environmental sequences analysis had revealed that Perkinsozoa, particularly the wider phylogenetic group that the pathogenic perkinsid from Georgia belonged to, are highly diverse and have been sampled from a range of freshwater environments and amphibian species [17].

The agent of severe Perkinsozoa infection has been primarily identified in tadpoles, although there are some reports of infection in adults [18]. Infection by Perkinsozoa is now considered an emerging disease and has been implied as responsible for die-offs of tadpoles throughout the USA, including populations of endangered species [4,16]. Using a targeted 18S rRNA approach, it has been demonstrated that additional diverse members of the freshwater Perkinsozoa clade, named “pathogenic Perkinsea clade” [16] or “novel alveolate group 01,” [17] can be detected from liver tissues from a wide diversity of Neobatrachia tadpoles and a range of disparate geographic locations. However, the relationship between disease and infection in this group is poorly established, and it is not yet clear if only the subclade that has been associated with mortality events across the USA is a disease-causing emerging parasite or whether the wider clade detected [16,17] is also associated with a cryptic disease. In addition, it has been hypothesized that disease symptoms may arise as a consequence of co-association of Perkinsozoa infections with other infectious agents and/or other forms of host stress [16,18].

## Are tadpoles infected with apicomplexans?

Approximately 50 species of the apicomplexan genus *Goussia* have been described, infecting a range of hosts, including marine fish and amphibian species (e.g., *Pelophylax* spp., *Rana dalmatina*, *R*. *temporaria*, *Bufo bufo*, and *Hyperolius viridiflavus*) [5]. Unlike apicomplexan *Eimeria* spp., which infect both tadpoles and adult frogs [19], *Goussia* spp. infections are restricted to the larval anuran life stages and appear to be lost during metamorphosis [20]. During infection, these parasites are located within the cytoplasm of enterocytes, while mature oocysts have also been observed inside melano-macrophages in the lumina of liver sinusoids (Fig 2B; [5]). The infected tadpoles show significant histopathological changes (e.g., disintegrating intestinal epithelium) and shed infectious oocysts in their faeces. However, there is no visible inflammatory response, no evidence of host mortality, and no disruption in the progression of metamorphosis [5], suggesting the pathology is not life-threatening. Thus, the parasitological and/or ecological significance of this infection in frog populations remains unquantified.

The second group of apicomplexan parasites shown to infect tadpoles is the subclass Gregarinasina represented by *Nematopsis temporariae*, a single species known to occur in tadpoles [6]. This microbe apparently forms intracellular infections of tadpole macrophages [6]. Gregarines are known to inhabit the intestine and other extracellular spaces of nearly every major group of invertebrates but were thought to be absent from vertebrates. Chambouvet and colleagues in 2016 showed that *Nematopsis* could be detected within the macrophages of *R*. *dalamatina*, *R*. *temporaria*, and *Hyla arborea* tadpoles (Fig 2B). It is interesting that both *Goussia* spp. and *Nematopsis* spp. are associated with and infect tadpole macrophages, although, in both cases, there is currently no evidence that either separate or joint infections by these protists result in disease. Despite this, the intracellular infection of macrophages suggests that these parasites may impede the tadpole immune system and may therefore impact host ecology.

## What outstanding issues remain?

The research summarized here demonstrates a range of alveolate infections of specific tadpole tissues and cell types critical for immune function and core physiology. The severe Perkinsozoa-infection etiologic agent associated with mass mortality events across the USA is now considered an emerging infection [16] and lies within a particular alveolate clade sampled from freshwater environments and tadpole tissues. We need to know how deterministic these infections are, either individually or in concert with other microbes and/or environmental factors, and the epizootiology of the disease. For example, environmental factors, such as pollution, can create sublethal stress resulting in suppression of the immune function, leading to an increase of disease susceptibility. As such, we need to apply an approach that allows the investigation of disease progression concordant with formal tests of Koch’s postulates [7]. We also need to know how virulence, if present, varies among different amphibian*-*associated perkinsozoans and apicomplexans.

It is also important to put these infections into a wider context and investigate if alveolates also infect salamanders and/or caecilians, of which members of both groups also have larval stages. An important additional question for future focus is to understand if these infectious agents occur in amphibian species commonly involved in meat or pet trade and whether farmed amphibians, such as bullfrogs, serve as reservoir hosts. If so, they potentially represent a threat through possible spillover into native and/or naïve amphibian populations and/or represent a risk of economic losses through infection of farmed amphibians. Furthermore, do infections and die-off events affect wider amphibian population structures, or are larval numbers typically weighted in such a way that early life history disease events are moderated? Studies that tackle these questions will be required in order to identify conservation threats and design appropriate mitigation strategies. We hope that this brief review will stimulate a community effort into understanding the biology of these infectious agents and the possible ecological impact of this infection on amphibians in natural ecosystems.

## References

[1] Wake DB, Vredenburg VT. Are we in the midst of the sixth mass extinction? A view from the world of amphibians. Proc. Natl. Acad. Sci. USA. 2008;105: 11466–11473. 10.1073/pnas.0801921105 18695221 PMC2556420

[2] Chanson J, Hoffmann M, Cox N, Stuart S. The state of the world’s amphibians Threatened Amphibians of the World. Stuart et al Barcelona/Gland/Arlington: Lynx Edicions/IUCN/Conservation International; 2008 pp. 33–52.

[3] Scheele BC, Pasmans F, Skerratt LF, Berger L, Martel A, Beukema W, et al Amphibian fungal panzootic causes catastrophic and ongoing loss of biodiversity. Science. 2019;363: 1459–1463. 10.1126/science.aav0379 30923224

[4] Davis AK, Yabsley MJ, Kevin Keel M, Maerz JC. Discovery of a novel alveolate pathogen affecting southern leopard frogs in Georgia: Description of the disease and host effects. EcoHealth. 2007;4: 310–317.

[5] Jirků M, Jirků M, Oborník M, Lukeš J, Modrý D. *Goussia* Labbé, 1896 (Apicomplexa, Eimeriorina) in amphibia: Diversity, biology, molecular phylogeny and comments on the status of the genus. Protist. 2009;160: 123–136. 10.1016/j.protis.2008.08.003 19038578

[6] Chambouvet A, Valigurová A, Pinheiro LM, Richards TA, Jirků M. *Nematopsis temporariae* (Gregarinasina, Apicomplexa, Alveolata) is an intracellular infectious agent of tadpole livers. Environ. Microbiol. Rep. 2016;8: 675–679. 10.1111/1758-2229.12421 27119160

[7] Fredericks DN, Relman DA. Sequence-based identification of microbial pathogens: a reconsideration of Koch’s postulates. Clin. Microbiol. Rev. 1996;9: 18–33. 8665474 10.1128/cmr.9.1.18 PMC172879

[8] Gray M, Miller D, Hoverman J. Ecology and pathology of amphibian ranaviruses. Dis. Aquat. Org. 2009;87: 243–266. 10.3354/dao02138 20099417

[9] Gower DJ, Doherty-Bone T, Loader SP, Wilkinson M, Kouete MT, Tapley B, et al *Batrachochytrium dendrobatidis* infection and lethal chytridiomycosis in caecilian amphibians (Gymnophiona). EcoHealth. 2013;10: 173–183. 10.1007/s10393-013-0831-9 23677560

[10] Martel A, Spitzen-van der Sluijs A, Blooi M, Bert W, Ducatelle R, Fisher MC, et al *Batrachochytrium salamandrivorans* sp. nov. causes lethal chytridiomycosis in amphibians. Proc. Natl. Acad. Sci. USA. 2013;110: 15325–15329. 10.1073/pnas.1307356110 24003137 PMC3780879

[11] O’Hanlon SJ, Rieux A, Farrer RA, Rosa GM, Waldman B, Bataille A, et al Recent Asian origin of chytrid fungi causing global amphibian declines. Science. 2018;360: 621–627. 10.1126/science.aar1965 29748278 PMC6311102

[12] Densmore CL, Green DE. Diseases of amphibians. Ilar J. 2007;48: 235–254. 10.1093/ilar.48.3.235 17592186

[13] Pasquier LD, Schwager J, Flajnik MF. The immune system of *Xenopus*. Annu. Rev. Immunol. 1989;7: 251–275. 10.1146/annurev.iy.07.040189.001343 2653371

[14] Marantelli G, Berger L, Speare R, Keegan L. Distribution of the amphibian chytrid *Batrachochytrium dendrobatidis* and keratin during tadpole development. Pac. Conserv. Biol. 2004;10: 173.

[15] Mikhailov KV, Janouškovec J, Tikhonenkov DV, Mirzaeva GS, Diakin AYu, Simdyanov TG, et al A complex distribution of elongation family GTPases EF1A and EFL in basal alveolate lineages. Genome Biol. Evol. 2014;6: 2361–2367. 10.1093/gbe/evu186 25179686 PMC4217694

[16] Isidoro-Ayza M, Lorch JM, Grear DA, Winzeler M, Calhoun DL, Barichivich WJ. Pathogenic lineage of Perkinsea associated with mass mortality of frogs across the United States. Sci. Rep. 2017;7. 10.1038/s41598-017-10456-1 PMC5579288 28860470

[17] Chambouvet A, Gower DJ, Jirků M, Yabsley MJ, Davis AK, Leonard G, et al Cryptic infection of a broad taxonomic and geographic diversity of tadpoles by Perkinsea protists. Proc. Natl. Acad. Sci. USA. 2015;112: E4743–E4751. 10.1073/pnas.1500163112 26261337 PMC4553764

[18] Landsberg J, Kiryu Y, Tabuchi M, Waltzek T, Enge K, Reintjes-Tolen S, et al Co-infection by alveolate parasites and frog virus 3-like ranavirus during an amphibian larval mortality event in Florida, USA. Dis. Aquat. Org. 2013;105: 89–99. 10.3354/dao02625 23872853

[19] Jirků M, Jirků M, Oborník M, Lukeš J, Modrý D. A model for taxonomic work on homoxenous coccidia: redescription, host specificity, and molecular phylogeny of *Eimeria ranae* Dobell, 1909, with a Review of anuran-host *Eimeria* (Apicomplexa: Eimeriorina). J. Eukaryot. Microbiol. 2009;56: 39–51. 10.1111/j.1550-7408.2008.00362.x 19335773

[20] Paperna I, Ogara W, Schein M. *Goussia hyperolisi* n. sp.: a coccidian infection in reed frog *Hyperolis viridiflavus* tadpoles which expires towards metamorphosis. Dis. Aquat. Org. 1997;31: 79–88.

